# Real-World Antifungal Therapy Patterns Across the Continuum of Care in United States Adults with Invasive Aspergillosis

**DOI:** 10.3390/jof10120876

**Published:** 2024-12-17

**Authors:** Barbara D. Alexander, Melissa Johnson, Mark Bresnik, Vamshi Ruthwik Anupindi, Lia Pizzicato, Mitchell DeKoven, Belinda Lovelace, Craig I. Coleman

**Affiliations:** 1 Infectious Diseases Division, Duke University, Durham, NC 27708, USA; barbara.alexander@duke.edu (B.D.A.); melissa.d.johnson@duke.edu (M.J.); 2 F2G Inc., Princeton, NJ 08540, USA; mbresnik@f2g.com (M.B.); belinda.lovelace@f2g.com (B.L.); 3 IQVIA, Falls Church, VA 22042, USA; ruthwik.anupindi@iqvia.com (V.R.A.); lia.pizzicato@iqvia.com (L.P.); mitch.dekoven@iqvia.com (M.D.); 4 School of Pharmacy, University of Connecticut, 69, North Eagleville Road, Storrs, CT 06269, USA

**Keywords:** invasive aspergillosis, antifungal agents, administrative claims, healthcare, therapy change

## Abstract

Changes to antifungal therapy (AFT) in invasive aspergillosis (IA) may occur due to intolerance, side effects, drug interactions, or lack of response. We describe AFT change patterns in IA patients. This was a US claims data study. IA patients were identified during the index hospitalization from October 2015 to November 2022. Patients were stratified by whether they ‘changed’ or ‘did not change’ AFT during or after the index hospitalization. AFT patterns were assessed for four lines of therapy or until loss of follow-up. First-line AFT began during the index hospitalization. Discontinuation with restart, modification, or switch in AFT ended the current line and initiated a subsequent line. Inverse probability-of-treatment weighting was utilized. Among 1192 adults with IA, 59.3% changed their AFT (60.0% modified AFT, 22.1% stopped first-line AFT and later initiated a new AFT for second line, and 18% immediately switched to a different AFT). Among those who changed AFT, triazole use predominated, with voriconazole (37.3–49.3%) and isavuconazole (19.3–26.7%) the most used across all AFT lines. Echinocandin use varied between 25.3 and 33.6% over all lines, and amphotericin B use increased over lines 1–4 (13.4–20.7%). Among the 40.7% of patients that completed AFT without changes, most received triazole monotherapy (62.8% voriconazole; 15.2% isavuconazole). Most patients required changes to their AFT.

## 1. Introduction

Invasive aspergillosis (IA) is a serious mold infection caused by *Aspergillus* species [1]. This opportunistic infection primarily affects individuals with weakened immune systems and is often associated with life-threatening complications as the fungus invades tissue at the primary site of infection (typically the lungs) and spreads to other organs.

According to treatment guidelines [2], early initiation of antifungal therapy (AFT) in patients with or suspected of having IA is strongly recommended. Voriconazole is often the first-line treatment of choice in IA patients, as it offers superior effectiveness and fewer adverse effects compared to amphotericin B deoxycholate. Alternatives such as isavuconazole, posaconazole, and lipid formulations of amphotericin B can be employed for patients with contraindications or intolerance to voriconazole. In severe or refractory cases, combination therapy, such as voriconazole with an echinocandin, may be considered.

Changes in AFT may be needed due to intolerance, adverse effects, drug interactions, or lack of response [3]. Prompt adjustment of AFT, tailored to the patient’s specific clinical situation and underlying conditions, is crucial for achieving optimal survival rates and reducing complications in affected individuals [2]. However, there is a paucity of data describing real-world AFT patterns evaluating adjustment and tailoring in patients with IA. Thus, we sought to characterize and compare real-world AFT patterns across the continuum of care among patients newly hospitalized with IA with a focus on those who changed or did not change their initial AFT.

## 2. Materials and Methods

### 2.1. Data Source

Claims data from IQVIA’s US New Data Warehouse from 1 January 2010–31 December 2022 were used [4,5]. The administrative claims dataset includes deterministically linked data from US Professional Fee and Prescription claims data and their hospital charge data master (CDM) database [4,5]. These data contain ~1 billion professional fee claims per year (representing >870,000 practitioners per month) with diagnoses, procedures, and office-administered drugs; ~1.6 billion retail or mail-order prescription claims representing dispensed prescriptions for about 85% of all pharmacies; and records from more than 450 hospitals (including inpatient and outpatient encounters within a facility with drug, procedure, diagnosis, and charge data for the entire stay along with patient demographics). This data capture insured patients across various payer types (e.g., commercial, Medicare, Medicaid).

The data were accessed in compliance with the Health Insurance Portability and Accountability Act. Institutional review board approval was not required for this retrospective analysis of the de-identified secondary data.

### 2.2. Study Design

This was a retrospective, observational cohort study of patients newly diagnosed with IA. To be included in this study, patients had to be ≥18 years old with ≥1 inpatient hospitalization claim in the hospital CDM database with an International Classification of Diseases, 10th Revision, Clinical Modification (ICD-10-CM) diagnosis code for IA (B44.0, B44.1, B44.2, B44.7) in the primary or non-primary billing code position during the patient selection period (1 October 2015–30 November 2022). The inpatient admission date for IA was defined as the index date (i.e., Time 0). Moreover, patients had to link to the professional fees and pharmacy claims data during the study period (1 January 2010–31 December 2022), have evidence of any systemic AFT for ≥3 days (any combinations of azoles, polyenes, or echinocandins) during the inpatient hospitalization, have activity denoted as ≥1 medical claim in the professional fee data set and ≥1 pharmacy claim in the pharmacy claims data set during the 6-month baseline period, and exhibit pharmacy stability (i.e., consistent reporting of data from the pharmacy most frequently visited by the patient during each month of the baseline period). Exclusion criteria included evidence of invasive fungal infections other than aspergillosis during the index hospitalization and missing patient gender.

Patients were subsequently stratified into two mutually exclusive patient cohorts, those who ‘changed’ or ‘did not change’ their initial AFT. AFTs were identified using National Drug Codes (NDC), Healthcare Common Procedure Coding System (HCPCS) codes, and hospital billing administration codes. Ffor medications identified using HCPCS codes or in the inpatient settings, days’ supply was imputed to one day (coding available from the corresponding author upon request). A ‘change in AFT’ was defined as the initiation of a new antifungal with or without discontinuing the first (i.e., discontinuation, switch, or modification defined as failure to refill an AFT within two times the days’ supply with restart of a non-first-line AFT; starting a new AFT within 30 days before or after discontinuation of prior AFT; or the addition or subtraction of AFT to their current regimen, respectively) at any point during the post-index period. Conversely, patients who ‘did not change’ AFT were defined as those who remained on their initial AFT, discontinued AFT without restart, or discontinued and restarted the same first-line antifungal over any period of time (the latter allowing ‘did not change’ patients to have more than one line of AFT).

AFT patterns were assessed until a patient was lost to follow-up (end of available data or death) or until four lines of therapy were identified (whichever came first). Initiation of an AFT (azole, polyene, or echinocandin monotherapy or in any combination) after the index date (Time 0) began the first line. Antifungals initiated within 3 days of the treatment initiation date were used to define the first line of therapy. ‘Changes’ (discontinuation with restart of a different antifungal, modification, or switch) to the first line of therapy ended that line and initiated the second line AFT. Subsequent changes to AFT lines initiated the start of the third line, and so on. Sankey plots showing the frequency of patients using various AFTs by line of therapy were created using the ‘networkD3’ package and R version 4.4.1 (The R Project for Statistical Computing, https://www.r-project.org/ accessed on 12 December 2024).

To control potential baseline differences between the AFT change and no-change cohorts, stabilized and trimmed inverse probability of treatment weighting (IPTW) was used to account for potential measured confounding between cohorts [6]. Propensity scores were calculated using logistic regression and transformed into weights for IPTW adjustment. Variables imbalanced at baseline were used to calculate the propensity score (i.e., age group, geographic region, payer type, year of index hospitalization, asthma, autoimmune conditions, bacteremia, cancer, chronic obstructive pulmonary disease, pneumonia, immunodeficiencies, myelodysplastic syndrome, neutropenia, transplant history, immunosuppressants, and total 6-month baseline healthcare costs). IPTW was used to estimate the average treatment effect (ATE) (i.e., expected causal effect of the treatment across all individuals in the population) and to create a weighted sample with a balanced distribution of measured covariates between cohorts. Cohorts were deemed well-balanced for a given variable if the standardized mean difference (SMD) between groups was <0.10. Analyses were conducted using SAS^®^ version 9.4 (SAS Institute Inc., Cary, NC, USA). A *p*-value of <0.05 was used to identify statistical significance between cohorts. No adjustments were made for multiple comparisons.

## 3. Results

### 3.1. Patient Selection and Characteristics

In total, 1190 patients were identified with IA. After IPTW weighting of this sample (which resulted in an effective sample size of 1192 IA patients), 707 (59.3%) patients changed, and 485 (40.7%) patients did not change AFT. Prior to IPTW, cohorts were imbalanced on many patient characteristics (Table 1). Characteristics in Table 1 with SMDs > 0.1 (i.e., age group, geographic region, payer type, year of index hospitalization, bacteremia, cancer, chronic obstructive pulmonary disease, immunodeficiencies, myelodysplastic syndrome, neutropenia, transplant history and complications, immunosuppressants, and total 6-month baseline healthcare costs) were deemed univariate predictors of AFT therapy change. After IPTW, cohorts were well-balanced (SMD < 0.1) on all characteristics except corticosteroid use in the 30 days prior to admission, which was marginally imbalanced with an SMD of 0.108. Patients identified were on average approximately 62 years old, were proportionally more male than female, primarily had Medicare or commercial insurance, and were mostly from the Western and Southern US regions (Table 1). The majority of patients had a Charlson Comorbidity Index of 3+ and the mean baseline costs prior to diagnosis were substantial (USD 183,656 to USD 188,619; 2023 USD).

### 3.2. Treatment Pattern Observations

In the change cohort, 100% of patients initiated a second line of AFT, 520 (73.5%) initiated a third line, and 343 (48.5%) initiated a fourth line (Table 2). In the no-change cohort, 148 (30.5%) discontinued and later restarted the same AFT (accounting for second-line therapy). Subsequent stops and restarts of the same AFT resulted in the third line (41 (8.5%)) and the fourth-line AFT (11 (2.3%)). Significantly more patients in the no-change cohort discontinued treatment across all lines of therapy (*p* < 0.05 for all) and discontinued without restarting in line 1 and line 2 (*p* < 0.05 for both) (Table 2). There were no differences in discontinuation and restart patterns in line 3 or line 4 between the change and no-change cohorts (*p* ≥ 0.05 for both).

Available post-index follow-up duration was 18 ± 21 months in the change and 13 ± 19 months in the no-change cohorts (*p* = 0.25). Length of time on AFT (days) was significantly shorter in the change cohort for the first line (13.7 ± 57.6 vs. 35.8 ± 91.8; *p* < 0.01) and the second line (26.4 ± 68.4 vs. 68.3 ± 130.3; *p* < 0.001) AFT compared to the no-change cohort. There were no differences in the length of time on AFT between cohorts for third or fourth line AFT.

**Figure 1:**
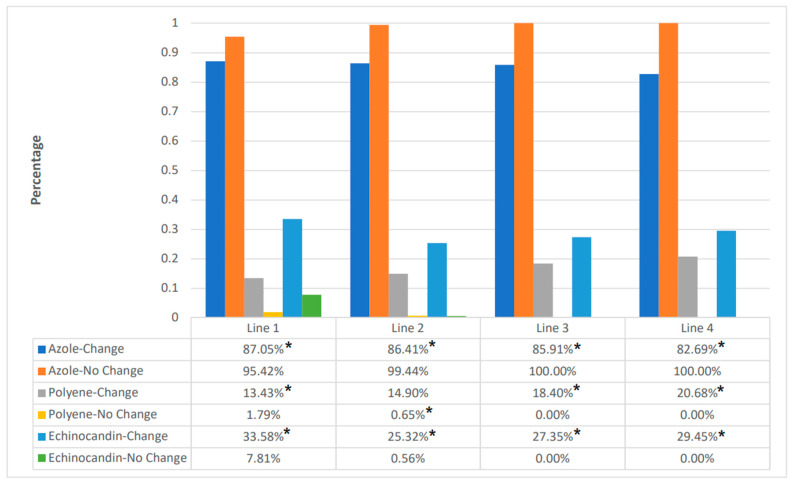
Percentage use of azole, polyene, and echinocandin antifungals, alone or in combination, as part of each line of therapy. * *p* < 0.05 for the change vs. no-change cohort comparison within the antifungal class.

Across all treatment lines, most patients (≥83%) received an azole, with fewer patients receiving an azole in the change cohort than the no-change cohort for lines 1–3 (*p* ≤ 0.01 for all) (Figure 1). More patients in the change cohort received secondary, or salvage, therapies consistent with IA treatment guidelines, including polyenes (for lines 1–3, *p* < 0.05 for all) (the preponderance as a lipid formulation) and echinocandins (for lines 1–3, *p* < 0.05 for all).

**Figure 2:**
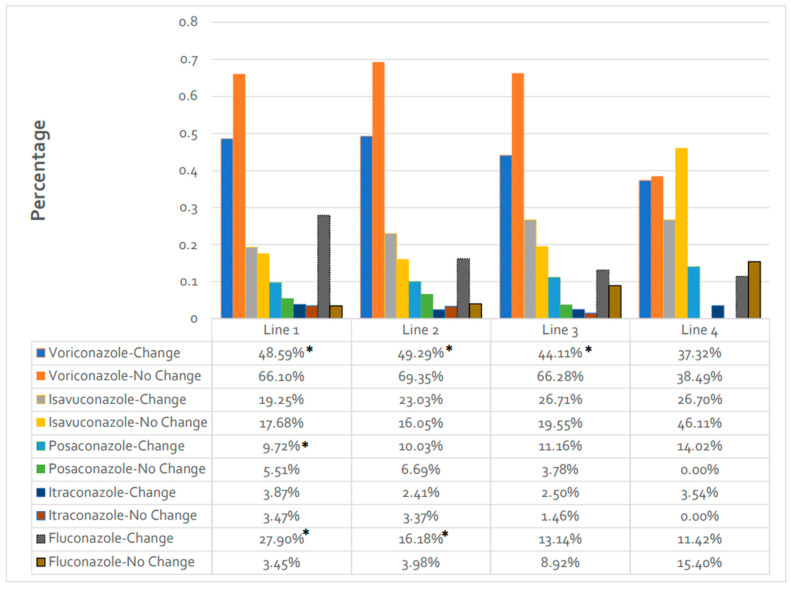
Specific azole antifungal use, alone or in combination, as part of each line of therapy. * *p* < 0.05 for the change vs. no-change cohort comparison within specific azoles.

Among azoles, the highest percentage of patients received voriconazole across lines 1–3 in both cohorts (44.1–69.4%); however, fewer patients received voriconazole in the change vs. no-change cohort (*p* < 0.05 for lines 1–3) (Figure 2). Isavuconazole was the second most utilized mold-active azole across lines 1–3 in both cohorts (16.1–26.7%). Multiple azole use was seen in 6.0–15.4% of patients in the change cohort vs. fewer than 0.8% in the no-change cohort across all lines.

**Figure 3:**
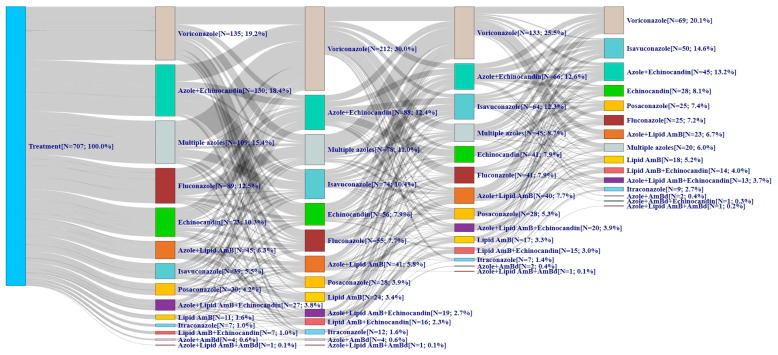
Treatment patterns among patients with invasive aspergillosis who changed antifungal therapy. AmB = amphotericin; AmBd = amphotericin deoxycholate; N = number. This Sankey diagram was used to visualize antifungal treatment sequences in patients with invasive aspergillosis who changed antifungal therapy. The blue bar represents the total number of patients evaluated. The subsequent bars reflect first, second, third, and fourth-line antifungal therapies used without regard to duration of treatment or timing of treatment change. The gray line represents the flow of patients from one antifungal therapy to another, with the thickness of the gray lines reflecting the number of patients.

**Figure 4:**
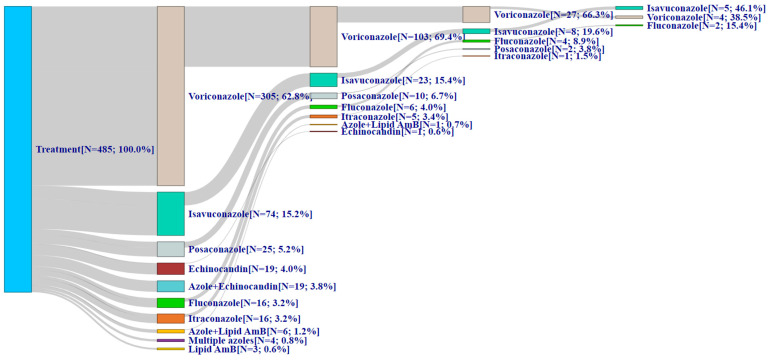
Treatment patterns among patients with invasive aspergillosis who did not change antifungal therapy. AmB = amphotericin; AmBd = amphotericin deoxycholate; N = number. This Sankey diagram was used to visualize antifungal treatment sequences in patients with invasive aspergillosis who changed antifungal therapy. The blue bar represents the total number of patients evaluated. The subsequent bars reflect first, second, third, and fourth-line antifungal therapies used without regard to duration of treatment or timing of treatment change. The gray line represents the flow of patients from one antifungal therapy to another, with the thickness of the gray lines reflecting the number of patients.

Just over one-half (54.3%) of patients who changed AFT began as first-line monotherapy, most commonly voriconazole (Figure 3). The remainder began with the first line AFT with a combination of drugs, with the most common combination used across all lines of therapy being an azole + echinocandin (12.4–18.4% across all lines). In comparison, monotherapy was used by nearly all patients (94.2–100% across all lines of therapy) in the no-change cohort (Figure 4). Most patients (62.8%) initiated first-line AFT with voriconazole monotherapy, and it remained the most common azole through the third line (isavuconazole was most common in line 4 at 46.1%). Very few patients in the no-change cohort were on combination therapy (5.8% of patients in line 1, <1% in line 2, and 0% in lines 3 and 4).

## 4. Discussion

To our knowledge, this is the first study to assess real-world treatment patterns of patients with IA in the US across their inpatient and outpatient treatment course using a nationwide claims database. Notably, about 60% of IA patients in this large cohort required a change in their initial AFT, supporting the notion that IA treatment is indeed complex, requiring multiple antifungal regimen changes. When comparing IA patients who required changes in their AFT versus those that did not, significant differences were observed. Patients who required AFT changes experienced shorter lengths of time on their first and second lines of treatment compared to those who did not require any changes. Among those that required a change in AFT, modification of the regimen (addition or subtraction of an antifungal agent) was more common than switching therapy. Conversely, >80% of patients that did not require an AFT change discontinued first-line therapy and did not restart another antifungal. The lack of AFT restart in the no-change cohort suggests patients successfully completed their course of AFT with their first-line regimen. Among both cohorts, discontinuation and later restart of the same AFT occurred throughout each of the lines of therapy. The reason behind this observation is unclear but may suggest that patients were experiencing adverse events, intolerability, or drug-drug interactions requiring a temporary hold of their AFT. Another possible reason may be problems with medication access (insurance coverage), which can occur during transitions of care or in the outpatient setting.

While azoles were the most common initial antifungal agents overall and remained the most common AFT through subsequent lines of treatment in both cohorts, patients in the change cohort were less likely to be on an azole across all lines of therapy versus those that did not change AFTs. Instead, patients in the change cohort were more likely to have received an echinocandin- or polyene-based therapy, not only in the first but also in subsequent lines of therapy, often as combination therapy. When a mold-active azole was used, voriconazole and isavuconazole (alone or with an echinocandin) were the most frequent in both cohorts. These patterns of azole use are consistent with current Infectious Disease Society of America (IDSA) clinical guidelines for IA, which recommend voriconazole as first-line azole therapy, and the results of the SECURE trial [7], which demonstrated isavuconazole to be non-inferior to voriconazole for the primary treatment of suspected invasive mold disease.

Some non-guideline-endorsed AFT was observed in our study. This includes monotherapy with echinocandins, dual azole regimens, and the use of the non-mold active azole, fluconazole. Each of these regimens occurred in the change and the no-change cohort. The use of fluconazole has been noted in a prior study of IA treatment patterns. In a real-world IA treatment patterns study performed by Henao-Martínez and colleagues [3], fluconazole was initiated in 11% of 3705 patients, although nearly one-half of these patients were switched off fluconazole in subsequent lines of therapy. The investigators suggested this was evidence of inappropriate fluconazole prescribing that was later corrected by fungal disease experts. While we agree that the observed use of fluconazole may represent mis-prescribing in some cases, it is also possible that some fluconazole was being used purposefully for prophylaxis (as suggested by its addition in later lines of therapy in our study) or that some use of fluconazole prophylaxis was captured prematurely as the first-line therapy due to the study’s dependence on prescription order/claims data. Temporary overlap occurring when fluconazole was being switched to a mold-active azole, or the addition of a mold-active azole to fluconazole, may explain some of the dual azole therapy seen in our study.

This study had several limitations. First, since we were using claims data, we identified patients with IA using diagnosis codes, and therefore misclassification of IA cases cannot be ruled out. Second, no explanation or clinical data (e.g., therapeutic drug levels, susceptibilities for *Aspergillus* isolates, etc.) were available to parse out reasons for initial selection or change of AFT. Studies are needed to better understand why patients change AFT (e.g., adverse events, intolerability, drug-drug interactions, ineffectiveness) and to identify multivariate/independent predictors of the need for AFT change (i.e., demographics, comorbidities, and the abovementioned clinical data). Finally, our study evaluated IA treatments between October 2015 and November 2022, and consequently, some of the prescribing patterns observed in this study may be temporally related to the index IA event (e.g., lower utilization of isavuconazole due to the study period predating clinical trials that demonstrated non-inferiority to voriconazole).

## 5. Conclusions

This contemporary snapshot of IA treatment patterns suggests the complexity of managing AFTs remains a major clinical challenge. Most patients with IA require multiple changes to their AFT. Factors contributing to the need to change AFT may include intolerances, toxicities, drug interactions, and resistant/refractory infections. Novel oral antifungals that reduce the need for AFT changes in this population are needed.

## Figures and Tables

**Table 1:** Baseline †* clinical characteristics of patients with invasive aspergillosis who changed vs. did not change antifungal therapy.

Clinical Characteristics	Pre-IPTW			Post-IPTW		
	Change (N = 708)	No Change (N = 482)	SMD	Change (N = 707)	No Change (N = 485)	SMD
Age group, n (%)						
18–44 years	101 (14.3%)	39 (8.1%)	0.35	84 (11.9%)	56 (11.5%)	0.02
45–64 years	300 (42.4%)	152 (31.5%)		270 (38.2%)	191 (39.3%)	
65+ years	307 (43.4%)	291 (60.4%)		353 (49.9%)	238 (49.1%)	
Gender, n (%)						
Female	300 (42.4%)	219 (45.4%)	−0.06	309 (43.7%)	225 (46.3%)	−0.05
Male	408 (57.6%)	263 (54.6%)		398 (56.3%)	260 (53.7%)	
Geographic region, n (%)						
Northeast	38 (5.4%)	46 (9.5%)	0.29	44 (6.2%)	38 (7.9%)	0.08
Midwest	40 (5.6%)	35 (7.3%)		52 (7.3%)	29 (5.9%)	
South	133 (18.8%)	127 (26.3%)		154 (21.8%)	106 (21.9%)	
West	497 (70.2%)	274 (56.8%)		457 (64.6%)	311 (64.2%)	
Payer type, n (%)						
Commercial	268 (37.9%)	155 (32.2%)	0.20	254 (36.0%)	176 (36.3%)	0.05
Medicaid	31 (4.4%)	14 (2.9%)		25 (3.5%)	21 (4.2%)	
Medicare	294 (41.5%)	247 (51.2%)		317 (44.8%)	218 (45.0%)	
Cash	0 (0.0%)	0 (0.0%)		0 (0.0%)	0 (0.0%)	
Unknown	115 (16.2%)	66 (13.7%)		111 (15.7%)	70 (14.5%)	
Year of index date, n (%)						
2015 (partial year)	20 (2.8%)	10 (2.1%)	0.18	18 (2.5%)	12 (2.5%)	0.05
2016	67 (9.5%)	43 (8.9%)		65 (9.3%)	44 (9.0%)	
2017	110 (15.5%)	68 (14.1%)		105 (14.8%)	71 (14.7%)	
2018	99 (14.0%)	57 (11.8%)		93 (13.1%)	57 (11.8%)	
2019	131 (18.5%)	75 (15.6%)		121 (17.2%)	88 (18.2%)	
2020	101 (14.3%)	86 (17.8%)		111 (15.8%)	75 (15.4%)	
2021	118 (16.7%)	83 (17.2%)		122 (17.2%)	87 (18.0%)	
2022 (partial year)	62 (8.8%)	60 (12.4%)		72 (10.1%)	50 (10.4%)	
CCI Categories, n (%)						
0	58 (8.2%)	46 (9.5%)	−0.05	60 (8.5%)	48 (10.0%)	−0.05
1	73 (10.3%)	59 (12.2%)	−0.06	82 (11.6%)	53 (10.9%)	0.02
2	140 (19.8%)	74 (15.4%)	0.12	130 (18.3%)	74 (15.3%)	0.08
3+	437 (61.7%)	303 (62.9%)	−0.02	436 (61.6%)	310 (63.9%)	−0.05
Comorbidities, n (%)						
Asthma	81 (11.4%)	67 (13.9%)	−0.07	88 (12.4%)	60 (12.4%)	0.00
Autoimmune conditions	39 (5.5%)	36 (7.5%)	−0.08	48 (6.7%)	33 (6.8%)	0.00
Bacteremia	49 (6.9%)	19 (3.9%)	0.13	40 (5.7%)	33 (6.8%)	−0.04
Cancer	329 (46.5%)	168 (34.9%)	0.24	294 (41.5%)	205 (42.2%)	−0.01
COPD	202 (28.5%)	217 (45.0%)	−0.35	246 (34.9%)	167 (34.5%)	0.01
Diabetes	235 (33.2%)	145 (30.1%)	0.07	235 (33.2%)	142 (29.2%)	0.09
HIV	16 (2.3%)	10 (2.1%)	0.01	15 (2.1%)	11 (2.2%)	0.00
Immunodeficiencies	177 (25.0%)	81 (16.8%)	0.20	154 (21.8%)	100 (20.7%)	0.03
Myelodysplastic syndrome	61 (8.6%)	16 (3.3%)	0.22	46 (6.6%)	37 (7.5%)	−0.04
Neutropenia	194 (27.4%)	55 (11.4%)	0.41	148 (21.0%)	104 (21.5%)	−0.01
Obesity	103 (14.5%)	71 (14.7%)	−0.01	101 (14.3%)	76 (15.7%)	−0.04
Pneumonia	328 (46.3%)	238 (49.4%)	−0.06	333 (47.1%)	231 (47.7%)	−0.01
Sepsis	175 (24.7%)	119 (24.7%)	0.00	166 (23.4%)	133 (27.4%)	−0.09
Transplant history	204 (28.8%)	85 (17.6%)	0.27	172 (24.3%)	124 (25.5%)	−0.03
Solid Organ Transplant	109 (15.4%)	61 (12.7%)	0.08	100 (14.2%)	69 (14.2%)	0.00
Bone marrow or stem cell transplant	100 (14.1%)	25 (5.2%)	0.31	76 (10.8%)	56 (11.6%)	−0.02
Transplant complications	147 (20.8%)	67 (13.9%)	0.18	124 (17.5%)	87 (17.9%)	−0.01
Solid organ transplant	85 (12.0%)	52 (10.8%)	0.04	77 (10.9%)	57 (11.8%)	−0.03
Bone marrow or stem cell transplant	63 (8.9%)	15 (3.1%)	0.25	47 (6.7%)	30 (6.1%)	0.02
Medications, n (%)						
Corticosteroids in prior 30 days	227 (32.1%)	138 (28.6%)	0.07	234 (33.1%)	137 (28.2%)	0.108
Immunosuppressants	154 (21.8%)	72 (14.9%)	0.18	131 (18.5%)	83 (17.1%)	0.04
Baseline All-Cause Costs, mean (SD)						
Total	USD 211,570 (USD 369,066)	USD 147,524 (USD 264,656)	0.20	USD 183,656 (USD 335,343)	USD 188,619 (USD 312,936)	−0.02

CCI = Charlson Comorbidity Index; COPD = chronic obstructive pulmonary disease; HIV = human immunodeficiency virus; IPTW = inverse probability of treatment weighting; SD = standard deviation; SMD = standardized mean difference. † Baseline period was defined as six months prior to the index date. * The following covariates were used to calculate propensity scores for inverse probability of treatment weighting: age group, geographic region, payer type, year of index, asthma, autoimmune conditions, bacteremia, COPD, pneumonia, immunodeficiencies, myelodysplastic syndrome, transplant history, immunosuppressants, cancer, neutropenia, and total healthcare costs. All covariates were well balanced as indicated by an SMD < 0.1 except corticosteroids, which were marginally imbalanced with an SMD of 0.108.

**Table 2:** Treatment patterns of patients with invasive aspergillosis who changed vs. did not change antifungal therapy.

Treatment Patterns	AFT Change								No AFT Change							
	Line 1		Line 2		Line 3		Line 4		Line 1		Line 2		Line 3		Line 4	
	n	%	n	%	n	%	n	%	n	%	n	%	n	%	n	%
N initiating each line	707		707		520		343		485		148		41		11	
Treatment patterns																
Discontinuation (n, %)	156	22.1% *	259	36.6% *	220	42.4% *	154	44.9%	408	84.1%	111	75.2%	35	84.5%	11	94.7%
With restart (n, %)	156	100% *	128	49.3% *	93	42.2%	86	55.6%	148	36.3%	41	37.1%	11	32.6%	7	66.2%
Without restart (n, %)	0	0% *	131	50.7% *	127	57.8%	68	44.4%	260	63.7%	70	63.0%	23	67.4%	4	33.9%
Switch (n, %)	127	18.0% *	79	11.1% *	51	9.8%	18	5.3%	--	--	--	--	--	--	--	--
Modification (n, %)	424	60.0% *	314	44.4% *	199	38.2% *	152	44.4%	--	--	--	--	--	--	--	--
Deletion (n, %)	277	65.3%	200	63.6%	134	67.2%	92	60.2%	--	--	--	--	--	--	--	--
Addition (n, %)	147	34.7%	114	36.4%	65	32.8%	60	39.8%	--	--	--	--	--	--	--	--
Days on treatment line																
Mean	13.7 *		26.4 *		33.4		45.5		35.8		68.3		51.2		54.6	
SD	57.6		68.4		89.2		122.4		91.8		130.3		104.5		91.1	

SD = standard deviation, AFT = antifungal therapy. *p*-values are calculated using weighted Chi-square tests for categorical variables and weighted *t*-tests for means. * *p* < 0.05 for AFT change versus no AFT change.

## Data Availability

Data are not publicly available. Data were licensed from IQVIA.

## References

[1] Thompson G.R., Young J.H. (2021). Aspergillus Infections. N. Engl. J. Med..

[2] Patterson T.F., Thompson G.R., Denning D.W., Fishman J.A., Hadley S., Herbrecht R., Kontoyiannis D.P., Marr K.A., Morrison V.A., Nguyen M.H. (2016). Practice Guidelines for the Diagnosis and Management of Aspergillosis: 2016 Update by the Infectious Diseases Society of America. Clin. Infect. Dis..

[3] Henao-Martínez A.F., Chastain D.B., Thompson G.R. (2023). Treatment pathways, switches, and inappropriate treatment during invasive pulmonary aspergillosis: Real-world experiences from a global research network study. Antimicrob. Agents Chemother..

[4] Niu X., Divino V., Sharma S., Dekoven M., Anupindi V.R., Dembek C. (2021). Healthcare resource utilization and exacerbations in patients with chronic obstructive pulmonary disease treated with nebulized glycopyrrolate in the USA: A real-world data analysis. J. Med. Econ..

[5] IQVIA Available IQVIA Data. https://www.iqvia.com/insights/the-iqvia-institute/available-iqvia-data.

[6] Austin P.C., Stuart E.A. (2015). Moving towards best practice when using inverse probability of treatment weighting (IPTW) using the propensity score to estimate causal treatment effects in observational studies. Stat. Med..

[7] Maertens J.A., Raad I.I., Marr K.A., Patterson T.F., Kontoyiannis D.P., Cornely O.A., Bow E.J., Rahav G., Neofytos D., Aoun M. (2016). Isavuconazole versus voriconazole for primary treatment of invasive mould disease caused by Aspergillus and other filamentous fungi (SECURE): A phase 3, randomized controlled, non-inferiority trial. Lancet.

